# Case of Hydrophobia

**Published:** 1812-12

**Authors:** 


*For the Medical and Physical Journal.*

CASE *of* HYDROPHOBIA, *by* DR. PINCKARD.

WILLIAM ROGERS, of Southampton-court, Queen-square, Bloomsbury, a strong healthy child, two years and a half old, was bitten, in the right arm, by a dog, on the 16th of July last. The wound was slight, and soon healed. The child went about as usual, there being no suspicion that the dog was mad: he was lying at the door, when the child, walking up the steps from the area, put his hand upon him, and he instantly bit him; upon which the father kicked the dog, and he ran away. No information regarding him has been since collected.

The child remained in his usual health, until Monday the 28th of September, when his parents perceived him to be unwell. He looked "rather pale," and they imagined that he had "head-ach." His mother put him early to bed, and he slept during great part of the night; but was occasionally "disturbed with starting and restlessness."

On Tuesday, September 29th, he was worse, and remained in bed. About three o'clock, his mother observed that he frequently grated his teeth: soon afterwards he seemed to be much agitated, had a wild look, and, as his mother expressed it, "made a noise in his throat or his stomach, as if trying to throw up something, but could not."

Mr. Hall, of Southampton-row, visited him in the evening, between nine and ten o'clock. The symptoms were then observed to be highly distressing, and somewhat peculiar; but it was not mentioned that he had been bitten by a dog, nor did any suspicion yet arise that his illness proceeded from such a cause.

His respiration was convulsive, and attended with a loud noise; but which, it was remarked, did not resemble the sound of the croup. He was restless, anxious, exceedingly agitated, and partially convulsed. When any thing was offered him to drink, he shuddered, and threw himself back.

Two drachms of ipecacuanha wine, with two grains of tartarized antimony, were administered, but scarcely any vomiting was produced. He threw off a small quantity of mucus, by a convulsive effort, and appeared to be sick, without having the power to vomit. A blister was applied to the thorax, and he was directed to take five drops of the tincture of squills, with the same quantity of ipecacuanha wine, frequently, during the night. Four grains of calomel were likewise given, which produced two evacuations before morning.

Mr. Hall repeated his visit at an early hour on Wednesday, Sept. 30th, when he found that the poor child had passed a most restless and distressful night. He had been extremely anxious and disturbed; breathed with a loud noise, as if flatus were constantly rising from the stomach and impeded in the œsophagus; had frequent grinding of the teeth; started in partial convulsions; and his parents said, that every time they "gave him any thing into his mouth, it evidently made him worse."

It was now mentioned, incidentally, that the child had been bitten, several weeks before, by a dog. Hearing this remark, Mr. Hall had no longer any doubt respecting the real nature of the disease.

At twenty minutes before nine o'clock I visited him. The child was then sitting upright, and supporting himself, with considerable firmness, on a woman's lap. His breathing was hurried and anxious, accompanied with a noise as if flatus were proceeding from the stomach, and producing a sense of suffocation, from his not having the power to expel it. His eye was widely opened, and the pupil much dilated. He looked distressed and terrified, and watched the objects around him with a peculiar quickness. Without falling into a strong fit of general convulsion, he was in constant motion and distortion, from what the woman who held him termed "inward convulsions," and which, she said, no one could judge of without feeling them, as she did, by having the child seated on her lap. His pulse was very feeble; his skin cold and clammy: he was sensible of what passed in the room; and his intellect seemed unimpaired. Repeated doses of antimony and ipecacuanha had been given to him without effect: wind escaped occasionally from his stomach, but he had no vomiting. He was anxious for drink, and asked eagerly for milk; but, when this or any other fluid was brought to him, he started back convulsed and shuddering, and could only take one hurried swallow, which he did with great agitation.

I poured some water, from a pitcher, into a basin before

 him, him, when he instantly shuddered, and threw himself back with a convulsive motion, looking terrified, the same as when he attempted to drink. A similar effect was produced on my sprinkling a few drops of water upon his arm, or his leg; or by agitating the air with my pocket handkerchief; also on moving the atmosphere about him, by opening and shutting the door. Feeling the current of air, he instantly shuddered, looked anxiously towards the door, and cried out "don't!"

Several times he asked impatiently for "more milk;" but the same feelings of distress were renewed every time it was brought to him: still, by a strong convulsive effort, he repeatedly took a single swallow, but immediately struggled as if to prevent suffocation, and was totally unable to drink by successive deglutition.

Another blister was applied to the upper part of the chest and the throat. He grew rapidly worse: the eye became prominent, the countenance assumed a livid hue, the breathing was more quick and hurried, and the agitation and partial convulsions increased. Twenty minims of tincture of opium were given to him in a small quantity of water, which he swallowed at one effort, in the same hurried manner, throwing himself back, shuddering and struggling as if from a terror of being suffocated. The convulsion was stronger than before, and he fell down backwards upon the woman's lap: a small quantity of frothy saliva issued from his mouth; he shut and opened his eyes in quick succession; the livid appearance of the countenance increased; the pulsation of the arteries became imperceptible; and he seemed to be expiring. Presently he was again sensible; but he now remained still, and quite free from convulsive distortion:—from extreme agitation he became suddenly composed; breathed quietly with a deeper inspiration; and, in twelve minutes, from the time of swallowing the last fluid, he died without a struggle or a sigh.

His eyes remained open, his mouth not quite closed, and so tranquil was the change, that neither the woman who held him, nor any of those who were standing by, could precisely mark the moment when he ceased to live.

It was about twenty minutes after nine when he expired. At three o'clock on the same day the body was opened by Mr. Harding, of Gower-street, in the presence of Mr. Hall, Mr. James Hall, Mr. Stacey, and myself. Scarcely any appearances indicative of disease were observable. In the cavities of the thorax and abdomen, the membranous surfaces were found to be unusually dry and clammy to the touch. The viscera were healthy. The stomach and intestines were distended

 with with flatus; and in the stomach was nearly a pint of fluid, with some coagulum of milk. The surfaces of the stomach, œsophagus, trachea, and larynx, were of healthy appearance. A very small spot at the posterior part of the pharynx, exhibited a slight fullness of the vessels, scarcely amounting to redness. In the course of the small intestines were two or three intus-susceptions, and, at a considerable distance below these, were seen a few spots of slight discoloration.

The same dryness or clamminess prevailed within the head, as in the cavities of the thorax and abdomen: the brain was firm in its texture, but of healthy appearance: the vessels of the pia mater were not much distended: scarcely any fluid was contained in the ventricles.

*Bloomsbury-square,*
*Nov.* 4, 1812.

